# The roles of plasticity *versus* dominance in maintaining polymorphism in mating strategies

**DOI:** 10.1038/s41598-017-15078-1

**Published:** 2017-11-21

**Authors:** Sylvain Moulherat, Alexis Chaine, Alain Mangin, Fabien Aubret, Barry Sinervo, Jean Clobert

**Affiliations:** 1 Station d’Ecologie Théorique et Expérimentale du CNRS (UMR5321), 2 route du CNRS, 09200 Moulis, France; 2 TerrOïko, 2 rue Clémence Isaure, 31250 Revel, France; 3 Institute for Advanced Studies in Toulouse, Toulouse School of Economics, 21 allée de Brienne, 31015 Toulouse, France; 4 Department of Ecology and Evolutionary Biology, Earth and Marine Sciences Building, University of California, Santa Cruz, CA 95064 USA

## Abstract

Although natural selection is expected to reduce variability, polymorphism is common in nature even under strong selective regimes. Discrete polymorphisms in mating strategies are widespread and offer a good opportunity to understand the genetic processes that allow the maintenance of polymorphism in relatively simple systems. Here we explored the genetic mechanism underlying the expression of discrete mating strategies in the rock-paper-scissors (RPS) game. Heterozygotes carry the genetic information for two different strategies, yet little attention has been devoted to the mechanisms underpinning heterozygote phenotype and its consequences for allele frequency dynamics. We explored the maintenance of polymorphism under 1) genetic dominance or 2) plasticity, as mechanisms driving the expression of alternative strategies in males. We developed an alternative mating strategy model and analysed allele frequency dynamics using time series analyses. Our results show that both genetic mechanisms can maintain polymorphism depending on population demographic characteristics but that plasticity can enhance the likelihood that polymorphism is maintained relative to dominance. Time series analysis on simulation outcomes show that the RPS game is mostly driven by a single strategy, but the importance of this strategy on long term dynamics is stronger when gene expression shows dominance rather than plasticity.

## Introduction

Evolutionary mechanisms such as natural selection and genetic drift are expected to reduce genetic variation within populations. Yet polymorphism is surprisingly widespread in animal and plant populations. To explain this paradox, a number of mechanisms have been proposed such as heterosis^1^, niche selection^2,3^ or negative frequency dependent selection (FDS)^4,5^ which are all known to allow the stable maintenance of polymorphism in natural populations^6^. Game theory suggests that multiple players competing for a resource (i.e. mates, nutrients) can be maintained by negative FDS in a system as long as no single player or strategy is an evolutionarily stable strategy^5,7,8^. In this context, allele frequencies can be stable or show frequency oscillations over time^7–9^. However, Trotter and Spencer^10^ showed that oscillations will be hardly detectable in natural conditions for more than three alleles. Discrete polymorphism such as trimorphism in mating strategies, which have been reported over the last two decades reviewed in^11^ are particularly interesting in this respect since they offer the opportunity to study the maintenance of polymorphism in a very simple and detectable context (i.e. control of mates and three discrete strategies). Indeed, in the discrete trimorphism context, the strategy pay-off matrix can form a rock-paper-scissors game (RPS game) between the three strategies with minor differences between empirical systems reviewed in^11^. In a RPS-type game, each strategy beats another one but is beaten by a third one. Theoretically, such a game can lead to monomorphic or polymorphic populations (stable equilibrium of strategy frequencies or oscillations over time)^4,7,8,12^. Empirical evidence for alternative mating strategies have been found in a variety of organisms including birds, lizards, damselflies, isopods, bacteria and loose-leaf flowers^4,13–18^, but the genetics underlying such polymorphism have received limited attention.

Heterozygotes carry genetic information for two strategies. An extensive literature exists on the genetic models for polymorphism^5,9,10,19–23^. However, only a few studies have attempted to explore the genetic mechanisms underlying the expression of alternative mating strategy games, usually assuming that a dominance relationship exists between alleles^8,21,23–26^. The assumption of genetic dominance is supported by several empirical examples advocating for dominance as a mechanism underpinning RPS games^17,26–28^ and is very common in genetic studies of morphological traits. On the other hand, empirical evidence for plasticity as a mechanism underpinning RPS games is relatively scarce either because this mechanism is rare or because it is more difficult to detect and therefore less often studied. However, plasticity is certainly possible and would translate into specific morph expression in heterozygotes or switches in individual reproductive strategies^4,24,29–31^ which could have important implications for fitness payoffs of each allele. Plasticity could be advantageous if the alleles coding for the different strategies are either neutral or synergetic in their respective expression^24^. However, this genetic information could be antagonistic and reduce a heterozygote’s performance compared to homozygotes. Such a cost would have an important impact the stability of the RPS game^7^ especially since a low cost to heterozygotes could induce a strong allele frequency disequilibrium which could explain why plasticity in alternative mating strategies has rarely been documented in nature to date. Regardless, very little theoretical attention has been devoted to the viability of phenotypic plasticity as a mechanism underpinning the expression of reproductive strategies in heterozygotes; especially in a context where the number of alleles is above two as in the RPS game context^9–11,26^.

The first goal of this study is to explore how different genetic mechanisms leading to the expression of alternative reproductive strategies and demographic stochasticity could influence the maintenance of trimorphism in RPS games under a range of initial conditions concerning species lifespan (short-lived to long-lived species) and allele frequencies (dimorphic populations, trimorphic populations and trimorphic with an allele at very low initial frequency). Indeed, demographic stochasticity occurs in most biological systems and has an important impact on population dynamics^32–34^ and game stability^7,35^ but is neglected by most studies of the maintenance of polymorphism^5,9,20,21,23,36^. In this regard, we performed population dynamic simulations of a virtual species playing a generic RPS game using an individual-based-model. The individual-based-model allows us to simulate the effects of individual genetic information (i.e. are individuals homozygotes or heterozygotes for the gene coding reproductive strategy?), social environment and demographic stochasticity on the maintenance of alternative reproductive strategies. In this framework, we explore the maintenance of polymorphism under two hypotheses on the mechanisms leading to individual phenotype:

- Genetic dominance: we assess genetic dominance of alleles coding for the mating strategy^24,26,28,31^. Past diploid models explicitly ignored over-dominance and only considered co-dominance or dominance^8,24,34^.
- Plasticity: heterozygotes are plastic and able to apply the best strategy given the current social context of that individual. Note that this could mean that an individual expresses a poor strategy that is the best available given its genes and the current social context or in a different context may mean the individual can express the best strategy overall relative to its neighbours.

The second goal is to explore the impact of the genetic structure and life history traits under each genetic hypothesis, on the maintenance of polymorphism given different assumptions about the strategies: (1) genetic expression basis of alternative reproductive strategies (2) costs linked to being a heterozygote.

To reach these goals, we built a stochastic individual-based model and used realistic assumptions that may generate cyclical structures in population dynamics (i.e. density dependence and RPS game). Even though the RPS game occurs in various ways (reviewed in^11^), we have chosen to implement the RPS game as a male alternative strategy game because it illustrates a simple game observed in natural populations of a few well studied species with a direct impact on individual reproductive performance and thus phenotype fitness^4,16^. The game assumes that M players are monogamous with a small home range, P are polygynous with medium home ranges and S are polygynous sneakers in P harems with large home ranges. Thus, M beats S and is beaten by P; P beats M and is beaten by S and finally, S beats P and is beaten by M ensuring the RPS game. Such a game is similar to the game extensively documented in *Uta stansburiana* and *Paracerceis sculpta* where M players resemble mate guarder males in *U. stansburiana* and the β female mimicking males in *P. sculpta*, P players would correspond to aggressive polygynous males in *U. stansburiana* and α mate guarder males in *P. sculpta* and S players to the female mimicking males in *U. stansburiana* and the γ criptic dwarf males in *P. sculpta*. We chose to implement the simplest possible genetic structure underlying our 3 phenotypes in the RPS game by using a single locus with three alleles corresponding to the genetic structure found in damselflies (*Ischnura elegans*) and the marine isopod (*P. sculpta*) (*m* = monogamous, *p* = polygynous, *s* = sneaker) leading to six genotypes (*mm, pp*, *ss*, *mp*, *ms*, *ps*)^16,17,28^. Hence, male reproductive strategies are determined as M, P or S depending on the genetic rule underlying the social strategy which we modelled in two ways:

- Allelic dominance (*p* > *s* > *m*) assumes that all genotypes containing a *p* (*pp*, *pm*, *ps*) allele lead to a P strategy, the M strategy exists when individuals have *mm* genotypes and all other genotypes (*ss*, *ms*) lead to a S strategy.
- Under the plasticity hypothesis, heterozygotes have the possibility to express both strategies provided for by their alleles. Our modeling approach therefore assumed a simple case where plasticity is generated by the differential expression of two alleles at a single loci, rather than by the differential expression of alleles at different loci (as is the case in plastic homozygotes). The strategy expressed will depend in this case on the frequency of each strategy in the population. New adults will adopt the apparently most optimal strategy in the population given their alleles and carry that strategy for the rest of their life (i.e. irreversible developmental plasticity). Thus, in a population where P and S are rare, an individual of genotype *pm* tends to adopt a P strategy rather than an M while the same individual in a population where P is rare but S is not will tend to adopt an M strategy.

In such a context we analysed simulation outputs using continuous wavelet analysis (time series analysis) which allowed us to 1) detect cyclical structures, 2) distinguish between cycles coming from population dynamics and cycles coming from the RPS game, and 3) identify causal relationships between phenotypes to understand how the genetic mechanisms underlying the expression of mating strategies affects the maintenance of polymorphism^37–39^.

## Results

### Stability of two strategy populations

Simulations with populations initially composed by individuals presenting only two of the three alleles leads to the fixation of the strongest strategy in the RPS game (i.e. P wins against M, M wins against S, and S wins against P). However, the time necessary for allele extinction depends on the genetic mechanism underlying the strategies. Indeed, the monogamous allele disappears faster than the polygynous and sneaker alleles under the genetic dominance hypothesis. In contrast, this same allele disappears more slowly under the plasticity hypothesis (see Supplementary Results).

Under the genetic dominance mechanism, a cost to heterozygote genotypes does not significantly change the probability or time before extinction of alleles. In contrast, under the plasticity hypothesis, the cost of being a heterozygote strongly influences the extinction speed of the *p* allele when P plays against S.

Whilst all simulations lead to mono-strategic populations, the speed at which this occurs presents high variation among strategies and is closely linked to population carrying capacity and demographic traits (i.e. adult and juvenile survival). Indeed, regardless of the losing strategy the global pattern is the same for a given set of parameters for carrying capacity and fecundity. The time to monomorphism increases with carrying capacity and decreases with fecundity (see Supplementary Figure S1).

### Maintenance of trimorphism

**Figure 1:**
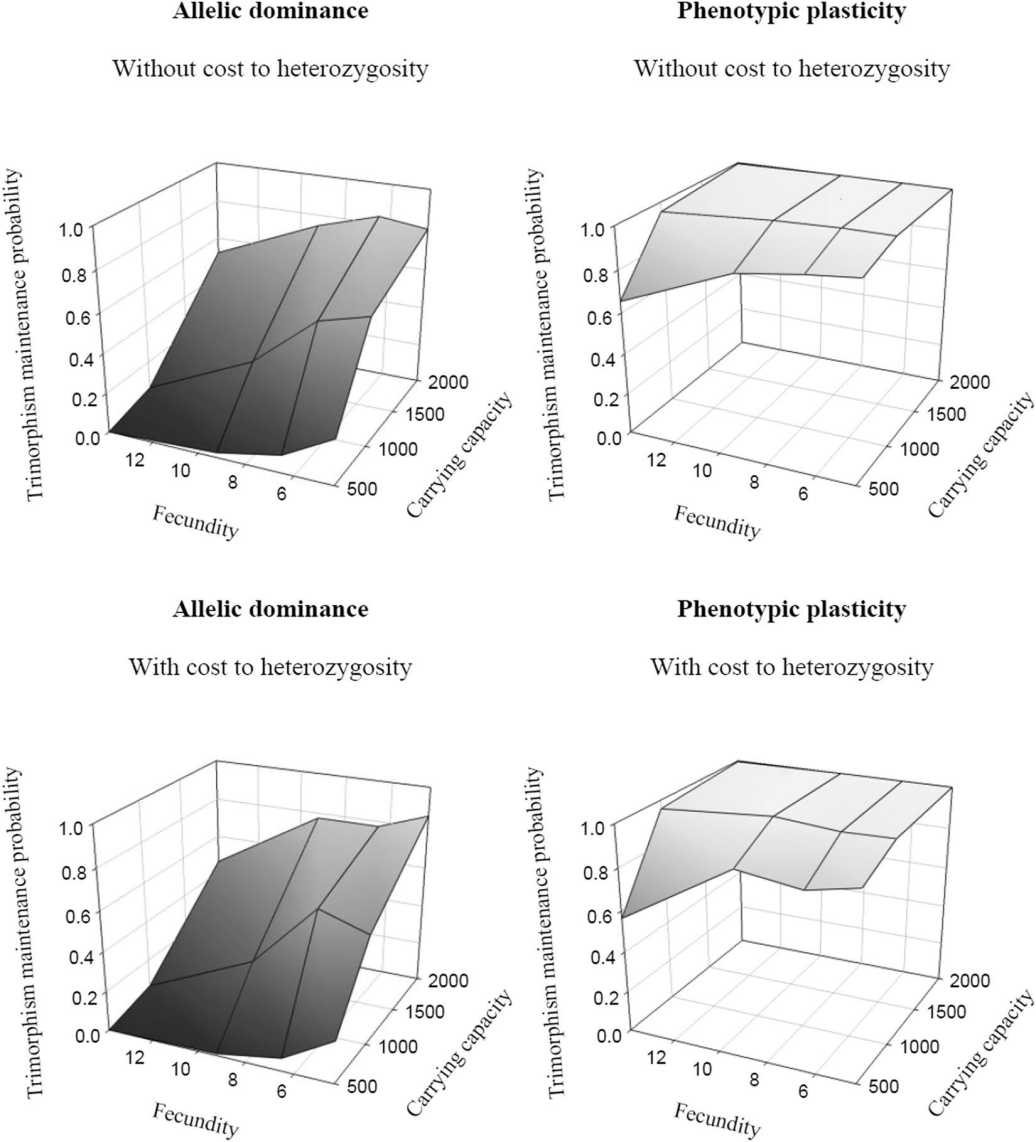
Trimorphism maintenance probability when males express alternative reproductive strategies under the allelic dominance or phenotypic plasticity hypotheses. The maintenance of the trimorphism is more easily maintained when individuals are plastic than if the phenotype depends on an allelic dominance relationship. The heterozygosity cost induces a low decrease of probability of maintenance of trimorphism (see text).

The underlying genetic mechanism has a strong effect on the maintenance of polymorphism. While the two genetic scenarios we modelled can both maintain polymorphism, alternative reproductive strategies are more easily maintained under the plasticity hypothesis than under the genetic dominance hypothesis (maintenance probabilities equal to 1 and 0.4 respectively) (Fig. 1).

Not surprisingly, when heterozygosity has a cost, the maintenance probability of trimorphism decreases. This reduction is low regardless of the genetic mechanism underlying reproductive strategies (Fig. 1). Under the dominance hypothesis, the reduced probability of maintaining the trimorphism when heterozygosity is costly seems to be explained by a change in average strategy frequencies. The P strategy frequency is similar to other scenarios but the S strategy frequencies fell from 0.4 to 0.3 thus increasing the risk of stochastic extinction. Under the plasticity hypothesis, the amplitude of strategy frequency oscillations was increased, thereby increasing occurrences of extinctions due to stochastic events at low frequency phases of a rare allele.

The trimorphism is more easily preserved when individual lifespan and carrying capacity increase, as one would expected given a reduction of stochastic extinction events as these parameters increase (Fig. 1). The life cycle also influences the probability that the trimorphism is maintained. Long lived species have an enhanced chance to produce rare offspring genotypes providing them with an advantage for their own reproduction. Indeed, when offspring are long lived, they have a possibility to “wait” for their genotype to become rare and therefore to enjoy a high selective advantage, hence reducing the extinction probability of their alleles compared to short lived species.

### Time series analysis of trimorphism frequency cycles

Regardless of the specific model configuration, wavelet analyses and multi-resolution analyses show that strategy frequency time series are structured on different time scales. Time scales of the different processes described above increase with increasing lifespan. For short-lived species (*S*_*a*_ = 0.2 and *S*_*j*_ = 0.2), the interpretable emerging structures are situated between 4 and 32 time steps (see Supplementary Figure S2). The fastest structure (a 4 time step period) is generated by density-dependence of recruitment. Indeed, these structures are independent of the alternative strategy interactions and found in the analysis of the population dynamic without alternative male mating strategies. The structures emerging from the alternative mating strategies occur at time scales between 16 and 32 time steps corresponding to the maximum correlation between pairs of strategy frequencies (detailed in Supplementary Figure S5). Moreover, strategies do not influence each of the other strategies at the same time scale. For example, the P strategy dynamic influences the M and the S strategies at a 16 time step scale whereas M and S strategies influence each other and the P strategy at a 32 time step scale (details in Supplementary Figure S5). Wavelet power spectra of the strategy frequency dynamic show that cyclical structures are detected at the 16 and 32 time scales (Supplementary Figure S5). The multi-resolution spliting confirms that these structures that make up the overall dynamic are cyclical (Supplementary Figure S5) and that the relative frequency of strategies exhibit a consistent pattern with those expected for the RPS game (see Supplementary Figure S2 for a detailed view of the pattern). A third category of structure exists at the 8 time step scale due to the link between the mating system and the density-dependence of recruitment (Supplementary Figure S5).

**Figure 2:**
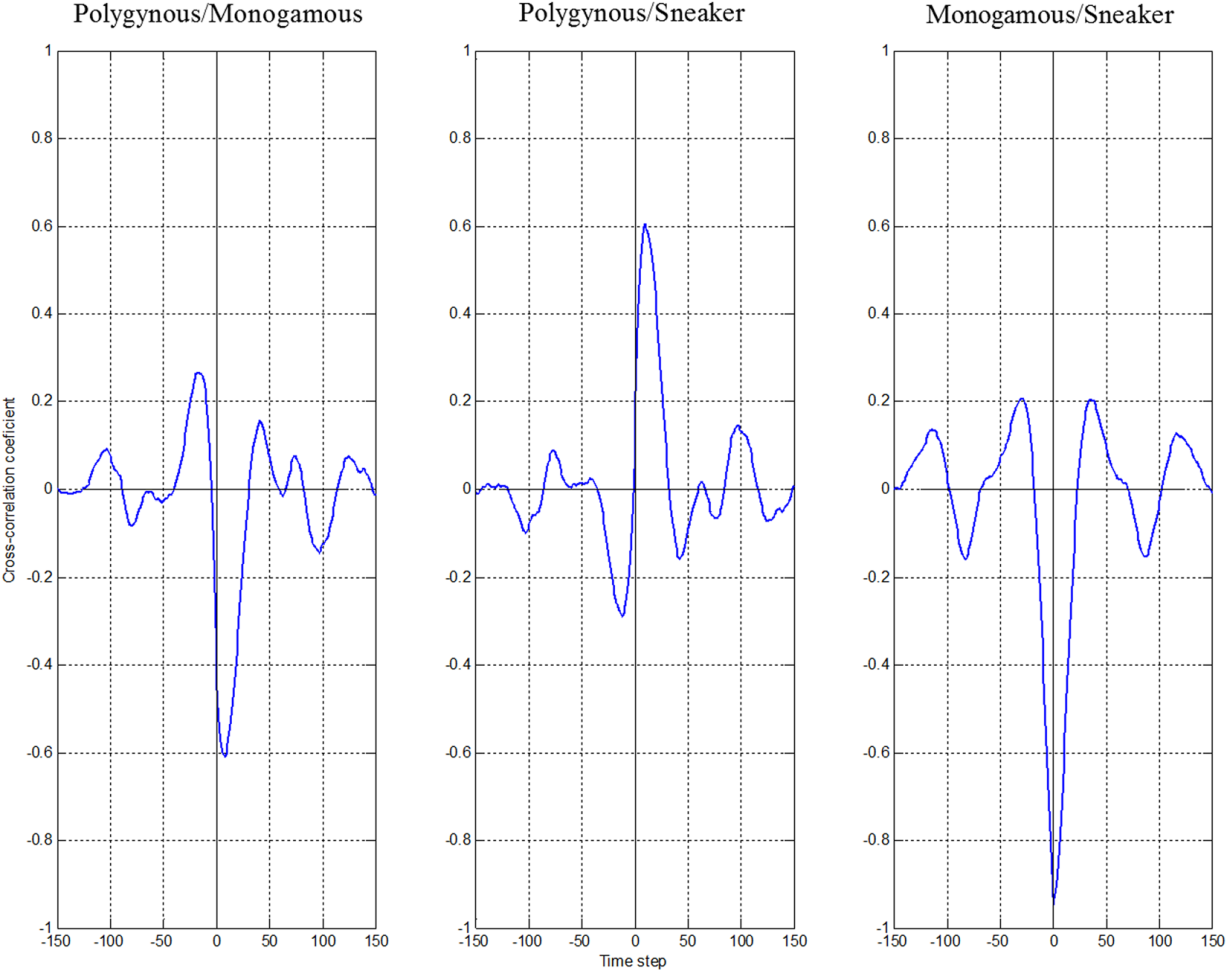
The influence that two time series impose on each other is detected by cross-corelation plots. If no causal relation exist between them, the cross-correlation function is symetric and centered on 0. The existence of a causal relation between the two series generates an asymmetry in the cross-correlation function^38^. Moreover, the direction of the deviation along the ordinate axis provides the direction of the correlation (see Supplementary Method for more details). Here, asymmetry and the direction of deviation in the cross-correlation function between the P strategy and the M strategy as well as between the P strategy and the S strategy, means that the P strategy has a negative effect on monogamous individuals and a positive effect on sneakers. In contrast, the cross-correlation function between the M strategy and the S strategy shows that the M strategy does not affect the allele frequency dynamic of the S strategy. At the global scale of the RPS game, the P strategy appears to drive the game.

The cross-correlation functions (see Supplementary Method) between frequencies of pairs of strategies show that the P strategy has a negative effect on the M strategy and a positive effect on the S strategy. The frequency of the M strategy does not influence the S strategy frequency suggesting that the game is largely driven by the P strategy (Fig. 2).

The gain functions^38^ between the number of individuals applying a strategy and total population size show that cyclical structures emerging from the alternative mating strategies are attenuated by demographic stochasticity (all gains < 1). The attenuation is more important under the plasticity hypothesis suggesting that the global demography is less constrained by the alternative mating strategies demographic outcome when strategies are plastic.

### Invasion of rare strategies

When rare, strategies differ in their probability of extinction. Indeed, under the genetic dominance hypothesis, S and M are unable to persist when rare, heterozygosity costs, carrying capacity or lifespan. In contrast, when the *p* allele is initially rare, the P strategy can invade and the probability of maintenance of the trimorphism converges to the same pattern as if all alleles would have been equally represented initially.

Under the plasticity hypothesis, both M and P strategies are able to reach high enough frequencies to allow for the maintenance of trimorphism while S cannot be maintained when initially rare. As under the genetic dominance mechanism, the pattern observed when *p* or *m* alleles are initially rare are similar to the pattern observed when the three alleles are equally represented. In this case, trimorphism maintenance probabilities are lower than in the case of equal frequency of all alleles (respectively reduced by 0.1 ± 0.01 and 0.3 ± 0.02) due to the enhancement of stochastic extinction of invading alleles.

## Discussion

### Genetic mechanism underlying frequency-dependent-selection and demographic stochasticity in trimorphic populations

As in previous studies, we find that discrete polymorphism can be maintained by negative FDS^7,8,40,41^. However, our results suggest that the underlying genetic control of the mating strategy phenotype strongly affects the probability that polymorphism is maintained. Previous genetic models, based on empirical work studying the properties of alternative mating strategies assumed that a dominance relationship existed in alleles coding for the three strategies^11,26,42^. However, even though trimorphism can be maintained under the allelic dominance hypothesis, the conditions for its maintenance are restrictive when compared to the plasticity hypothesis. Indeed, inclusion of demographic stochasticity under the genetic dominance hypothesis will likely lead to a loss of one of the three alleles underlying the alternative mating strategies phenotypes, especially when compared to the plasticity hypothesis. This would be especially important since evolution of a third strategy will most likely require some length of time where the two other strategies are stable^8^.

From our results, we should expect that the trimorphism will be maintained by dominance and negative frequency dependent selection only in large populations or in long-lived species with high dispersal rates, although these are precisely the cases where trimorphisms are apparently rare^11^, or in small populations of a metapopulation as suggested by Corl *et al*.^12^. However, the expression of a phenotype may affect individual survival. Polygynous males of the side blotched lizards and highly harassed females of *I. elegans* have reduced survival^30,43^. Thus we may expect that the difference in survival rate depending on the expressed strategy will change the observed pattern of phenotype frequencies in the field in different ways. First, the oscillation frequencies of the affected phenotype may increase with the reduction of survival. Second, the phase function between strategies may be affected, thus modulating the sequence of high frequencies of each phenotype from a highly structured sequence to chaotic sequences. These two last changes of the observed pattern linked to differences in survival rates of the expressed phenotype would be closely linked to the payoff matrix and to the interaction between demographic processes (survival) and the game between strategies (payoff matrix). In these two cases, oscillations due to frequency dependent competition between strategies may be undetectable in the field and the use of wavelet analysis would allow disentangling the demographic components from the game component and their interaction in the maintenance of the alternative strategies. A third possible consequence of survivorship tied to strategy expression is that the oscillation in strategy frequencies may disappear and tend towards stable frequency equilibrium of the three strategies where the frequency of each strategy will depend on the payoff matrix design. Indeed, this result has also found in other theoretical studies^8,10^ where oscillation of the phenotype frequencies can disappear and tend to stasis depending on the payoff matrix design, especially with a genetic dominance mechanism. By contrast, our results show that phenotypic plasticity can help maintain strategy cyclicity over longer time scales relative to genetic dominance (see also^44^ for an analogous scenario in female mate choice). Together, our results suggest that cyclicity among phenotypes of polymorphic species in natural populations might be maintained by 1) strategy plasticity in heterozygotes, 2) demographic stochasticity when genetic dominance occurs, or 3) some external factor such as predation that shifts strategy frequencies off their stable equilibrium.

When heterozygotes are able to adopt the best strategy with respect to their social environment, polymorphism can be maintained regardless of the population size or the species life cycle. The plasticity hypothesis (or a heterozygote advantage) seems to allow the maintenance of polymorphism for a wider range of species life strategies and environments; and accordingly we might expect the plasticity mechanism to be more wide-spread in trimorphic species and by extension to more complex systems with more than three alleles and more continuous and less detectable polymorphisms. In this respect, previous empirical as well as theoretical studies have shown that heterozygote advantage often underpins the maintenance of polymorphism of reproductive strategies in natural populations^1,24,36,45–47^. However, in contrast of the dominance hypothesis which benefits from experimental evidence in damselflies^17,28^, our plasticity hypothesis is only suggested by indirect evidence from one specific case. Indeed, in the *U. stansburiana*, there is evidence that *ms* color genotype, *sp* color genotypes and *ss* color genotypes exhibit plasticity as modulated by components of the endocrine system (testosterone for *ms;* ^30^, and the interaction of Follicle stimulating Hormone and Leutenizing Hormone for *ms*, *sp* and *ss* ^29^.

Numerous ways of modelling the genetic mechanisms underlying the reproductive strategies are possible, we restricted this study to just 2 of those possible mechanisms. Indeed, the genetic dominance mechanism we implemented assumed that *p* > *m* > *s* which is just one of the 6 possible combinations of dominance relationships that could exist with 3 alleles. However, additional simulations (not shown) with different combinations of dominance relationships (*p* > *s* > *m* and *s* > *p* > *m*) led to similar general results in terms of the conditions for the maintenance of polymorphism. Furthermore, these additional explorations tend to suggest that the maintenance of polymorphism relies more on the payoff matrix design than the dominance relationship within alleles. Additional simulations using different genetic mechanisms such as codominance, heterozygote advantage, or different forms of plasticity (e.g. pleiotropy) would help determine if the genetic structure or payoff matrix play a bigger role in determining the maintenance of polymorphism in general.

Further, our modeling approach assumed a simple case where plasticity was generated by the differential expression of two alleles at a single loci, rather than by the differential expression of alleles at different loci (as is the case in plastic homozygotes for instance), warranting further and more complex modeling on the topic.

### Time series analysis and cyclical system functioning

Theory says that in the RPS game, all strategies must have equivalent global fitness over long time scales to constitute an ESS^7,42^. This would imply that the maintenance of the cyclical structures generated by the RPS game may be equally sensitive to frequency variation of the three strategies. Our time series analyses have shown that, in the general case, the genetic dynamic emerging from the RPS game is driven by a single strategy, the P strategy. This suggests that even though all the strategies have equivalent global fitness, the system will be more sensitive to perturbation of the P strategy and that trimorphism is closely linked to the frequency dynamic of the P strategy. Whether this result holds for empirical systems and if different systems have the same strategy as the driver of cyclical dynamics remains to be seen when the methods we outline here are applied to empirical data.

In conclusion, the model we developed allowed us to test the possibility that two mechanisms of genic expression (genetic dominance or phenotypic plasticity) may maintain polymorphism. We found that, plasticity more easily maintained strategy polymorphism and was more likely to maintain frequency oscillations in a negative FDS system. Hence, both mechanisms are able to maintain polymorphism but, in the wild, phenotypic plasticity may be more widespread due to a less restrictive set of conditions leading to polymorphism maintenance. The coupled use of simulation and time series analysis enlightened the causal relationships which may drive simple negative FDS processes and by extension probably more complex genetic structures and associated polymorphisms. This approach also highlighted that in a specific case, discrepancies between empirical observations and theoretical models may not be only attributed to model simplification. Indeed, the lengths of field time series are too short in most of the cases to permit the disentangling between the social game cyclical structures and demographic ones. As a consequence, while the studies of most empirical systems do not provide sufficiently long time series to investigate the influence of each strategy on population frequency dynamics, the time series analysis techniques used here suggest that social game pay-off structure may not reflect the current importance of each social strategy when demographic stochasticity and density dependence are present.

## Methods

### Model design

#### Nomenclature

**Figure 3:**
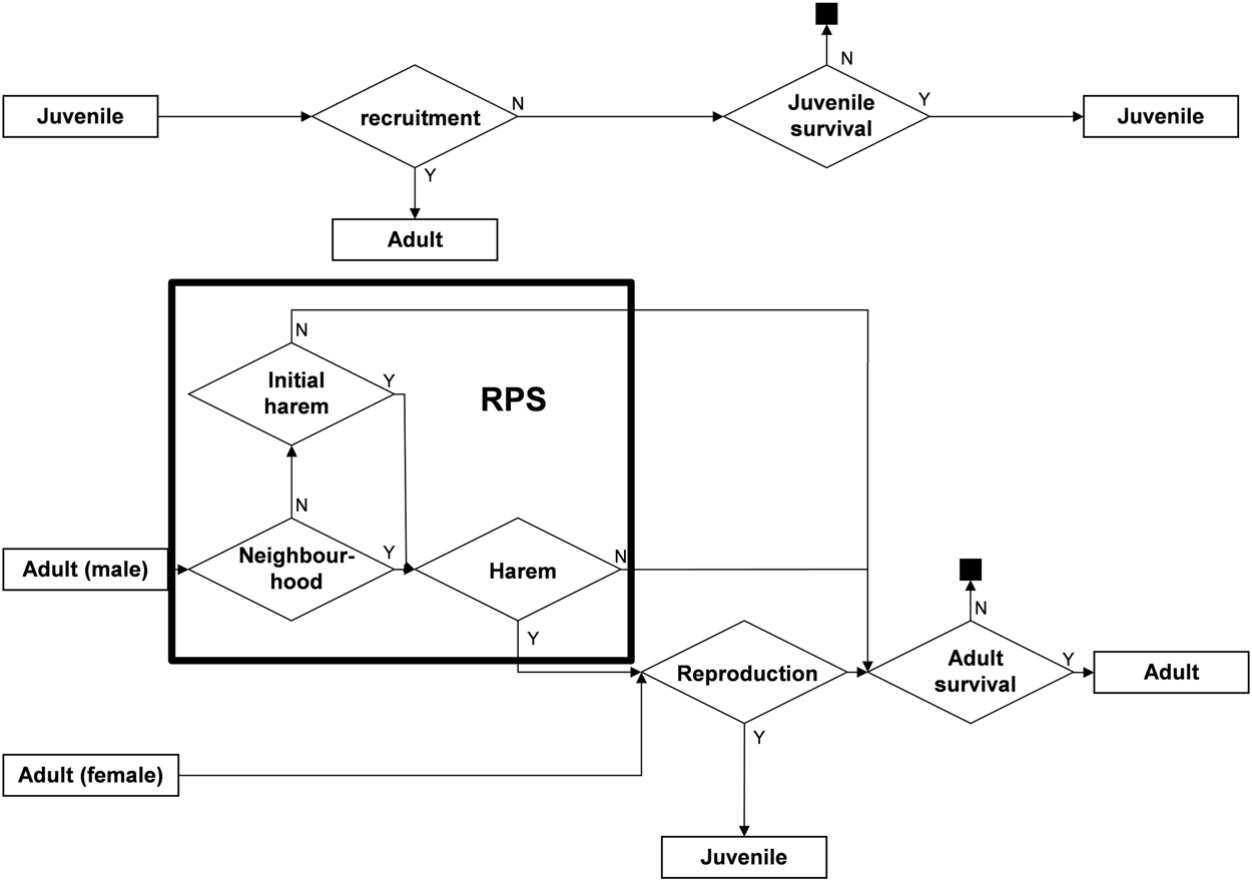
Life history of the model during a single time step showing the transitions between age classes (rectangles) and decisional rules (rhombus) regulate these transitions. For example for a juvenile, if in the recruitment phase (recruitment rhombus) the result is ‘yes’ (Y), it becomes an adult and the individual enters the cycle for adults and then begin the mating phase. If the result is ‘no’ (N), it stays in the juvenile cycle and will be tested for its survival. The black squares correspond to death of an individual that is removed from the simulation. The mating phase is composed by the three processes in the bold rectangle and includes the implementation of the RPS game.

*Population dynamic model description:* The model is a basic stochastic demographic model of two sexes and two age classes (reproductive and non-reproductive individuals) which can model the population dynamics of any species (fauna or flora) with multiple reproductive status classes and sexual reproduction. The demographic process is divided in four main phases (Fig. 3, Table 1):

1. Juvenile recruitment: juveniles are randomly recruited into the adult population depending on population density. The non-recruited juveniles survive depending on the juvenile survival probability (*s*_*j*_).
2. Mating: males and females are mated through male RPS game rules depending on the neighbourhood composition and mating strategies.
3. Reproduction: offspring are produced according to the genotype of their parents
4. Adult death: adults survive according to the adult survival probability *s*_*a*_.

We assumed that a scramble competition exists among all age classes and that our populations ($N$) are growing with a realistic asymptotic growth rate $\lambda \backsim 1.1$ ^32^. The competition coefficient $k$ was fitted for population carrying capacities ($K$) of 500, 1000 and 2000 individuals. We designed 4 life cycles based on the variation of survival in juveniles ${s}_{j}$, and adults ${s}_{a}$, and fecundity *F* leading to a gradient of short-lived species to long-lived species (see Table 1).

### RPS game modelling

The home range size reflects the number of neighbours each male has and thus, the number of competitors for mates. To estimate at each time step the relative average fitness of each strategy playing against the beaten strategy (i.e. P *vs* R, R *vs* S, S *vs* P), we modelled the negative FDS by designing a 3 × 3 pay-off matrix ${W}_{1}$ following equation 1 which corresponds to the average fitness gain of the strategy *i* playing against *k* where *f* are the frequencies of strategies *i* and *k* and ${W}_{0}$ the absolute dyadic pay-off matrix (i.e. gain of an individual of a *i* strategy playing in a world of only *k* strategy, Table 1).

1 $${W}_{1(i,k)}={f}_{i}\sum _{1}^{k}{f}_{k}{W}_{0(i,k)}$$

To determine the impact of a putative cost in heterozygotes expressing their phenotype on the maintenance of trimorphism we implemented a cost (*c*) to heterozygotes. We considered that a game between heterozygotes is equivalent to a game between pure strategists but that pure strategist had an advantage playing against heterozygotes which was implemented as an increased probability to win by a factor *c*. We also assumed that the initial heterozygote mean harem size was reduced by a factor *c* as was the probability of winning in the RPS game against a pure strategist.

During phase 2 of the demographic model, the mating process is implemented in three steps (Fig. 3): First, a male’s neighbourhood is determined. The neighbourhood size is assumed to follow a Poisson distribution and neighbours are randomly allocated among other males regardless of their strategy. Then, each male of each strategy constitutes their harem. Harem size also follows a Poisson distribution and females are allocated randomly to a harem (i.e. once a female is allocated to a male, it cannot be allocated to another one at that stage of the simulation). The constitution of harems begins with P males, then M males, and finally S males. Next, the contests between males starts with S playing against P neighbours, then M playing against S neighbours and finally P playing against M neighbours. In this game phase, each pair of males plays the game where the winning strategy has a probability of acquiring each female of the losing male’s harem prior to copulation according to a Bernouilli event. The average winning probability of the focal male depends on the frequency of each strategy within the current population. Hence, two players with the same phenotype have the same chance of winning the female (*p*_*win*_ = 0.5) while a player expressing a different strategy from its opponent has an additional gain or loss to the chance of winning the contest depending on the relative strategy frequencies in the current population and determined using equation 1. For example, a P strategiest in a population where M is common will have an increased chance of winning. Then each female won from a neighbour is added to the winner’s harem and removed from the loser’s harem. So while males can usurp females from another male, no mixed paternity of a single female’s reproductive output exists since reproduction occurs after final harems are composed.

### Runs

Under each genetic mechanism, we examine a range of possible life history trait values provided in Table 1. For each combination we considered several initial conditions of allele frequencies.

1. We performed runs with only two alleles in the population to determine how long it took for each strategy to outcompete the other.
2. We aim to determine if the genetic mechanism allows maintenance of reproductive strategy trimorphism. Consequently, we assumed that all alleles are initially equally represented in the population.
3. We simulated the “appearance” of a third allele (and therefore a third strategy) in a two allele population. Appearance of the new allele is implemented by exchanging 20 heterozygote individuals (carrying alleles for the two initial strategies) into 20 heterozygotes who carry the new allele and the allele coding the strategy that is currently losing. For example, in a population with only ‘*p*’ and *‘m*’ alleles, we removed 20 ‘*pm*’ individuals and replaced them by 20 ‘*ms*’.

**Table 1:** Nomenclature and default parameter value used in the different models.

Parameters, variables and their distributions	Value(s) of parameter for general model		Description		
**Demography**					
$\sigma$; Binomial	0.5		sex ratio		
${s}_{j}$; Binomial	0.2, 0.5		Juvenile survival		
${s}_{a}$; Binomial	0.2, 0.5		Adult survival		
k	0.004, 0.002, 0.001		competition coefficient		
$F$; Poisson	14, 9.2, 6.5, 4.4		fecundity		
**RPS game**					
Male game					
w_0_		**P**	**M**	**S**	RPS game pay-off matrix
	**P**	1	2	0.50	(Mean number of females won by a focal male of a given strategy (by row: polygynous (P), monogamous (M) or sneaker (S)) when play against 3 other males of the same strategy (by columns))
	**M**	0.5	1	2	
	**S**	2	0.5	1	
*h*_*p*_; Poisson	3				Polygynous harem size
*h*_*m*_; Poisson	1				Monogamous harem size
*h*_*s*_; Poisson	0.5				Sneaker harem size
*n*_*p*_; Poisson	3				Number of polygynous male neighbours
*n*_*m*_; Poisson	3				Number of monogamous male neighbours
*n*_*s*_; Poisson	3				Number of sneaker male neighbours
Heterozygosity cost					
c	0.2				Cost factor

Simulations are run 300 time steps which are usually long enough to reach stability of the population dynamics and genetic structure. All runs are repeated 200 times. To perform time series analysis, simulations leading to a dynamic equilibrium of the 3 strategies are run again on 2050 time steps to provide sufficiently long time series to ensure time series analysis robustness.

### Time series analysis

No time series analyses are performed for simulations exploring costs of plasticity for the heterozygote phenotypes due to the impossibility of maintaining trimorphism during enough time steps. RPS games are likely to show cyclical dynamics due to the interactions between strategies reviewed in^11^. To identify and characterize the temporal structure emerging from the RPS game, we perform a wavelet analysis using “Morlet” wavelet^48,49^. Wavelet analysis has multiple applications in ecology such as studying, interactions between climate change and phenology^50^, switches in biological cycles due to anthropogenic pressures^51^, spatial behaviour and distribution of species^52–54^, and various other applications reviewed in^48^. Wavelet analysis detects when information *sensu* Shannon^55^ is present and how this information “travels” across temporal scales^48,56,57^. The technical benefits of such analysis in ecology more generally are reviewed by^48^ but in our study, wavelet analysis allows us to identify if a cyclical structure exists in the time series and when it occurs. The method allows us to determine the periodicity of a cyclical structure in a continuous space even if the process is non-stationary (i.e. the periodicity changes). Thus, wavelet analysis allows for the characterization of slowing or accelerating cycles or punctual structures^48^ in contrast to discrete methods such as Fourier analysis that focus on stationary cyclical structures. We then perform multi-resolution analysis to identify the time scale of patterns emerging from the RPS game^38^. Multi-resolution analysis assumes that the global signal is the result of the addition of multiple signals at different temporal scales and thus separates the global signal into its different components^38,39^. Multi-resolution analysis allows us to analyse interactions between different time series signals and we used this analysis to identify interactions between time series emerging from the RPS game due to different strategies. Finally we use cross-correlational analysis between strategies to identify the causal nature of changes in allelic frequency^48,58,59^. In this statistical context (signal processing context), cross-correlational analysis between the time series frequencies of two different phenotypes is a measure of similarity of two series as a function of different time lags of one cycle relative to another cycle. The cross-correlation is similar in nature to the convolution of two functions (see^38^ for mathematical details of the method). In other words, this last analysis answers the following question: can some strategies drive the cycles of others or are cycles due to some other effect?

Modelling and time series analyses are performed under the MATLAB^®^ environment using the Wavelab toolbox^60^.

## Electronic supplementary material

Supplementary information
