# Supplementary material for: The roles of plasticity versus dominance in maintaining polymorphism in mating strategies
Source: Sci Rep. 2017 Nov 21;7:15939. doi: 10.1038/s41598-017-15078-1 (PMC5698437; doi:10.1038/s41598-017-15078-1)
Supplement: Supplementary file 1 — Supplementary information [file 41598_2017_15078_MOESM1_ESM.pdf]

The roles of plasticity *versus* dominance in maintaining polymorphism in mating strategies.

Sylvain Moulherat, Alexis Chaine, Alain Mangin, Fabien Aubret, Barry Sinervo, Jean Clobert

Supplementary Results: Extinction probability and strategy extinction time in two phenotype populations

Simulations with populations initially composed of individuals presenting only two of the three alleles leads to the fixation of the strongest strategy in the RPS game context (i.e. P wins against M, M wins against S and S wins against P) (Figure S1).

Under the genetic dominance hypothesis, the loss of *m* alleles is higher compared to other alleles and the *m* allele persists roughly three times longer than the *s* and *p* alleles do (Table S1). This difference between the *m* allele and the others alleles can be explained by the fact that in a polygynous/monogamous population, heterozygotes carry the *m* allele but express the winning P phenotype whereas in the other heterozygous cases, individuals express the losing strategy (i.e. *ps* and *ms* individuals express respectively losing phenotypes P and S) increasing allele extinction speed.

Under the plasticity hypothesis, the trend is reversed and the *m* allele disappears faster than the *p* and *s* alleles (Table S1). Loss times for *p* and *s* alleles were similar to *m* under the genetic dominance assumption due to the ability of heterozygotes to express the winning strategy. As expected, extinction time of the *m* allele decreased under the plasticity hypothesis because *pm* individuals can adopt a strategy of low selective value. In addition, the losing M strategy can be sometime chosen whilst genetic dominance leads to the P strategy winning in all cases. However, this decrease may also be explained by the asymmetry of mating systems between polygynous and monogamous strategists[1]. Such asymmetry may accelerate the extinction speed of *m* allele compared to others.

Under the genetic dominance hypothesis, the heterozygote cost does not significantly change the probability or time before extinction of alleles (Table S1). Under the plasticity mechanism, the heterozygote cost only influences the extinction speed of the *p* allele when P plays against S (Table S1). Under this hypothesis, *p* and *m* alleles have similar extinction patterns explained by the matting system asymmetry. Indeed, in these cases, *ps* individuals have reduced harem size compared to *pp* and *ss* individuals.

Figure legends:

Figure S1: Under the RPS game assumptions, populations composed of only two phenotypes leads to the fixation of a single phenotype according to the RPS game rules. In this case, P wins against M, M wins against S and S wins against P. The graphs represents the number of time steps leading to the extinction of the losing strategy assuming allelic dominance *p>s>m* (upper panels) and that heterozygotes express the best strategy (lower panels). From left to right, eviction time of *s* allele when playing against M strategists, loss

time of *m* allele in a P/M population and eviction time of *p* allele when the P strategy is sneaked by S strategists.

Figure S2: Construction by multi-resolution analysis of the three strategy frequencies in the RPS game context at time scales for which we detected cyclical structures (4 time step to 32 time step scale). Paper strategy corresponds to the red lines, rock corresponds to the blue lines and scissors corresponds to the black lines. The pattern exhibited by strategy frequencies between 16 and 32 time steps scale is consistent with the pattern expected for an RPS game [2-4]. Structures exhibited at the 4 time step scale corresponding to density-dependence of population dynamics and the structures of the 8 time step time scale is a mixture of density-dependence and the RPS game.

Table and table legend:

Table S1: Under the RPS game assumptions, populations composed of only two phenotypes leads to the fixation of a single phenotype according to the RPS game rules. In this case, P wins against M, M wins against S and S wins against P. The Table reports the number of time steps before extinction of the disappearing strategy in a population with two phenotypes (i.e. extinction of *p* allele in a population of P and S strategists). Alleles are assumed to be carried by a single locus and $p > s > m$.

**model hypothesis**	**genetic dominance**			**plasticity**		
**allele**	*p*	*m*	*s*	*p*	*m*	*s*
**mean extinction**	27	130.4	34.9	157.7	69.7	154.5
**SE extinction time**	1.8	11.6	2.5	10.8	5.2	12.1
**heterozygosity cost**						
**mean extinction**	27.3	127.2	32.5	79.9	70.3	152.3
**SE extinction time**	1.9	11.4	2.3	5.2	5.5	12.5

Figure:

Figure S1:

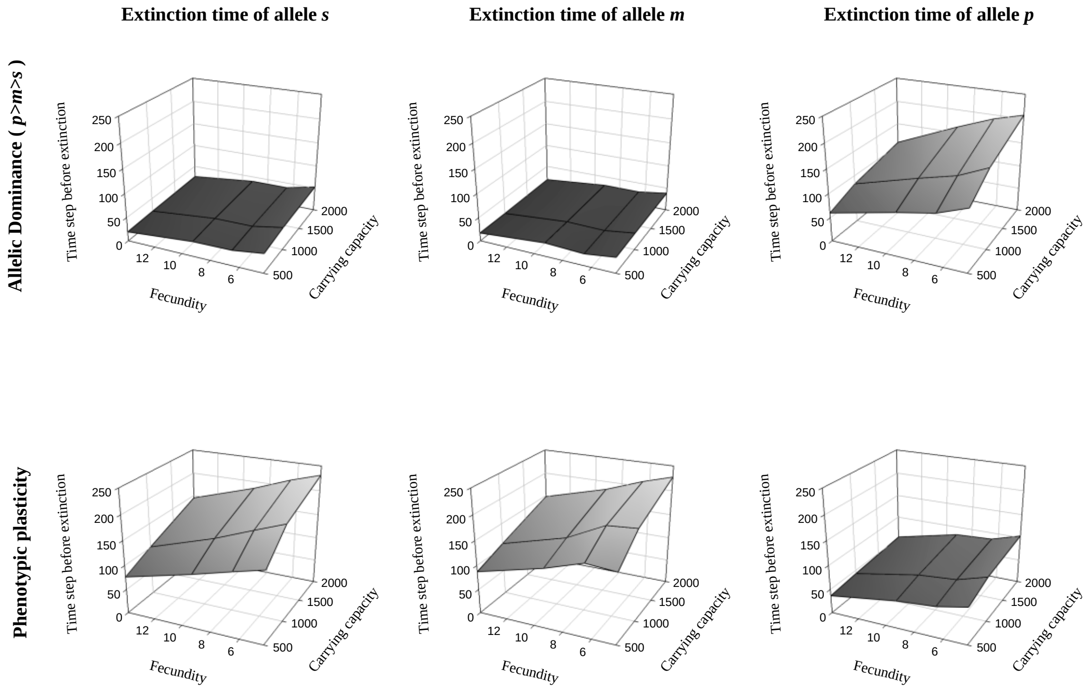

Figure S2:

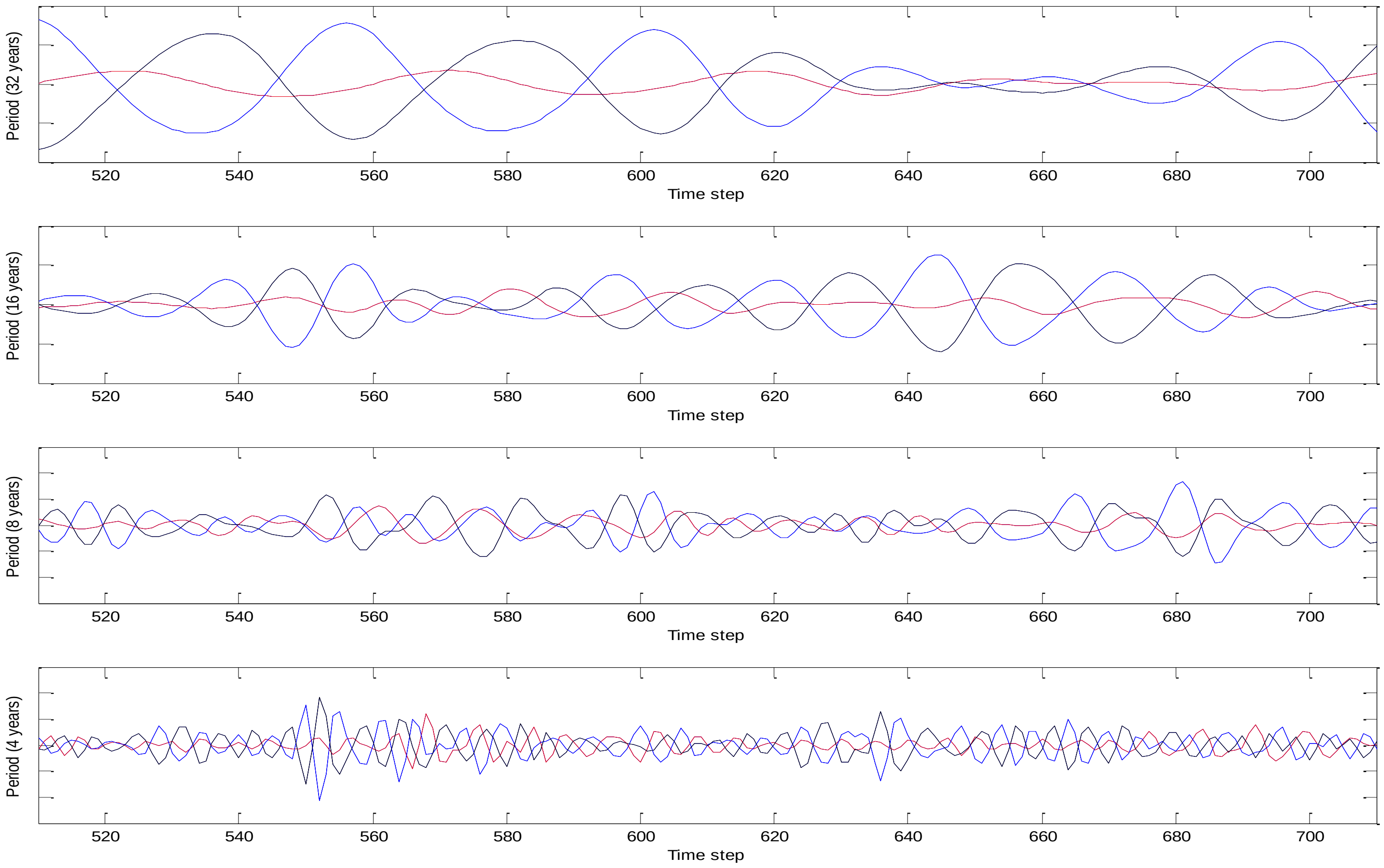
Period (32 years)
520
540
560
580
600
620
640
660
680
700
Time step
Period (16 years)
520
540
560
580
600
620
640
660
680
700
Time step
Period (8 years)
520
540
560
580
600
620
640
660
680
700
Time step
Period (4 years)
520
540
560
580
600
620
640
660
680
700
Time step

Supplementary Method: Elements of time series analysis using continuous wavelet

The goal of this supplement is to explain the major concepts underlying wavelet and multi-resolution analysis rather than all the mathematics and statistics which are well established see [5,6-10]. In essence, we provide a mini-primer on these analyses which are less familiar to evolutionary ecologists.

Wavelet and single signal multi-resolution analyses

Here we examine wavelet analysis and multi-resolution signature of a simple sinusoidal function. We built three simple sinusoidal functions (f_1, f_2 and f_3) made of 10400 points. Function f_1 (equation S1) presents an abrupt change in oscillation frequency, f_2 (equation S2) is composed of two sinusoidal functions of different amplitude and period and f_3 (equation S3) presents a slow-down in its oscillations (Figure S3):

$$\begin{cases} f_1(t) = A_1 \sin\left(\frac{2\pi t}{p_1}\right) & if\ t < t_t \\ f_1(t) = A_1 \sin\left(\frac{2\pi t}{p_2}\right) & if\ t \geq t_t \end{cases} \quad \text{(equation S1)}$$

$$f_2(t) = A_1 \sin\left(\frac{2\pi t}{p_1}\right) + A_2 \sin\left(\frac{2\pi t}{p_2}\right) \quad \text{(equation S2)}$$

$$\begin{cases} f_3(t) = A_1 \sin\left(\frac{2\pi t}{p_1}\right) & if\ t < t_t \\ f_3(t) = A_1 \sin\left(\frac{2\pi t}{p(t)}\right) & if\ t \geq t_t \end{cases} \quad \text{(equation S3)}$$

Where A_1=2, A_2=1, $p_1=\pi/60$, $p_2=\pi/12$, t_t=5000 and $p(t) = p_1 + (p_2 - p_1)\left(\frac{t-at_t}{bt_t}\right)$ with a=0.9 and b=0.2.

We then perform a wavelet analysis using the "Morlet" wavelet [11] and a multi-resolution analysis on the modeled signals (Figure S3). The wavelet analysis determines the temporal localization of information carried by the signal. This analysis detects when information [12] is present and how this information "travels" across temporal scales[9-11]. Multi-resolution analyses then assumes that the global signal is the result of the addition of the signal carried by the different temporal scales and separates the global signal into its different components [6,7].

Figure A1 shows the wavelet and the multi-resolution signatures of the three functions:

- f_1: The wavelet power spectrum shows that a high power spectrum (in dark red) is moving-down from low frequency oscillations (period ≈ 20 temporal unit or t.u.) to medium frequency oscillations (period ≈ 8 t.u.). The multi-resolution analysis shows that the signal is composed of a single signals at period = 16 t.u. until t = 5000 t.u. where it switches to another single signal at period = 8 t.u.
- f_2: The wavelet power spectrum shows that high power of spectrum is carried by two different temporal scales (period ≈ 20 t.u. and period ≈ 8 t.u.) all along the signal. The multi-resolution analysis shows that the signal is composed of a mixture of two signals with at period = 16 t.u. and period = 8 t.u.
- f_3: The wavelet power spectrum shows that a high power spectrum is located around the 8 t.u. time scale until the signal shows an amplified oscillation. Then the high power spectra moves-up to lower frequencies of oscillations. The multi-resolution analysis shows that the signal is initially composed of a single signals at period = 8 t.u.. Then, the global signal becomes a combination of signals from period = 4 and sliding to 16.

Multiple signal analyses

In this section, the main idea is to determine if a signal (S_1) influences another signal (S_2) and if so, to determine the temporal scale of this influence. The cross-correlation function between S_1 and S_2 allows us to determine if a causal relation exists between S_1 and S_2 see [6 for details]. If no causal relation exists, the cross-correlation function is symmetrical whereas it is asymmetrical if a causal relationship exists. When the function is asymmetrical, if both the signals are in phase, the function is centered on 0, otherwise the function is centered on the phase difference. The direction of the deviation from 0 reflects the direction of the relationship. If S_1 is positive and induces a positive response of S_2, the deviation will be also positive. Finally, the cross-correlation function presents at the same time the effect of S_1 on S_2 ($x>0$) and the feed-back effects of S_2 on S_1 ($x<0$) [6].

With complex signals, it can be interesting to identify the time scale at which one signal, S_1 or its components influences S_2 (Figure S4). We have seen before that multi-resolution tools can split the signal into its different components and we are able to perform correlations between S_1 or its components and S_2's components to detect the time scale of interactions between S_1 (or its components) and S_2 (or its components). The intensity of these correlations corresponds to the intensity of the transfer of information from S_1 to S_2 [6].

Figure A3, shows the combined used of the previously described statistical technics on our simulation results.

References:

Abarbanel HDI, 1996. Analysis of Observed Chaotic Data. New York: Springer-Verlag.

Box GEP, Jenkins GM, 1976. Time Series Analysis, Revised ed. San Fransisco: Holden-Day.

Cazelles B, Chavez M, Berteaux D, Menard F, Vik JO, Jenouvrier S, Stenseth NC, 2008. Wavelet analysis of ecological time series. Oecologia 156:287-304. doi: 10.1007/s00442-008-0993-2.

Jenkins GM, Watts DG, 1968. Spectral Analysis and its Applications. San Fransisco: Holden-Day.

Mandelbrot B, 1997. Fractales, hasard et finance: Flammarion.

Mari J-L, Glangeaud F, Coppens F, 1997. Traitement du Signal pour Géologues et Géophysiciens. Paris: TECHNIP.

Maynard-Smith J, 1982. Evolution and the theory of games. Cambridge: Cambridge University Press.

Meyer Y, Roques S, 1993. Progress in Wavelet Analysis and Applications. Gif-sur-Yvette: Frontieres.

Shannon CE, 1948. A Mathematical Theory Of Communication. Bell System Technical Journal 27:379-423.

Sinervo B, Calsbeek R, 2006. The developmental, physiological, neural, and genetical causes and consequences of frequency-dependent selection in the wild. Annual Review of Ecology Evolution and Systematics 37:581-610. doi: 10.1146/annurev.ecolsys.37.091305.110128.

Sinervo B, Lively CM, 1996. The rock-paper-scissors game and the evolution of alternative male strategies. Nature 380:240-243.

Figure legends:

Figure S3: Wavelet analyses and multi-resolution analyses of three simple sinusoidal functions (left panels) with an abrupt change in oscillation period (f_1), a function (f_2) composed of two sinusoidal functions and a function (f_3) beginning regularly and at mid-time accelerating and then slowing-down. Central panels are the wavelet power spectra of the different signals and represent the specific signature (see text). The multi-resolution (right panels) presents the specific signatures of the different functions in a discrete environment. Signals are split into time scale specific sub-signals. For readability, only temporal windows between 4000 t.u. and 6000 t.u., temporal components of oscillations between 4 t.u. and 32 t.u. are represented and the multi-resolution analysis have been normalized.

Figure S4: Correlation between a signal S_1 and the components of a signal S_2. S_1 is the frequency of the M strategy and S_2 is the frequency of P strategy under the genetic dominance mechanism without cost of heterozygosity and both the sexes exhibiting alternative reproductive strategy. The maximum of information transfer from S_1 to S_2 occurs at the 32 time steps scale and corresponds to the maximum correlation between S_1 and each components of S_2.

Figure S5: Wavelet ("Morlet" wavelet) and multi-resolution analyses of strategy frequencies dynamics. From top to bottom, results are provided for focal time series of respectively Paper, Rock and Scissors strategy frequency dynamics. The time series are outputs from a simulation with allelic dominance and where heterozygosity is not costly. Left panels correspond to the correlation between the non focal time series of strategy frequencies and the multi-resolution decomposition of the focal time series. Dark blue, black and red lines represent the effect of each of the two non-focal time series on the component of the focal time series listed on the left of the graph and grey, orange and light blue lines are the effects of the focal time series on each of the non-focal time series. Non-focal time series are red, blue or black for Paper, Rock and Scissors strategy frequency dynamics respectively and are orange, light blue and grey respectively when considering the effect of focal time series on non-focal time series. Central panels are the power spectra analysis of the strategy frequencies. The colors code for power values from relative low values (dark blue) to relative high values (dark red). The nature (period, stationarity) of the cycles detected

in the time series are determined using their specific signatures of alternation between red and blue regions. Right panels are the multi-resolution decomposition of the strategy frequencies which correspond to the time series decomposition at each time scale. The analyzed time series is the sum of the multi-resolution decomposition.

Figures:

Figure S3

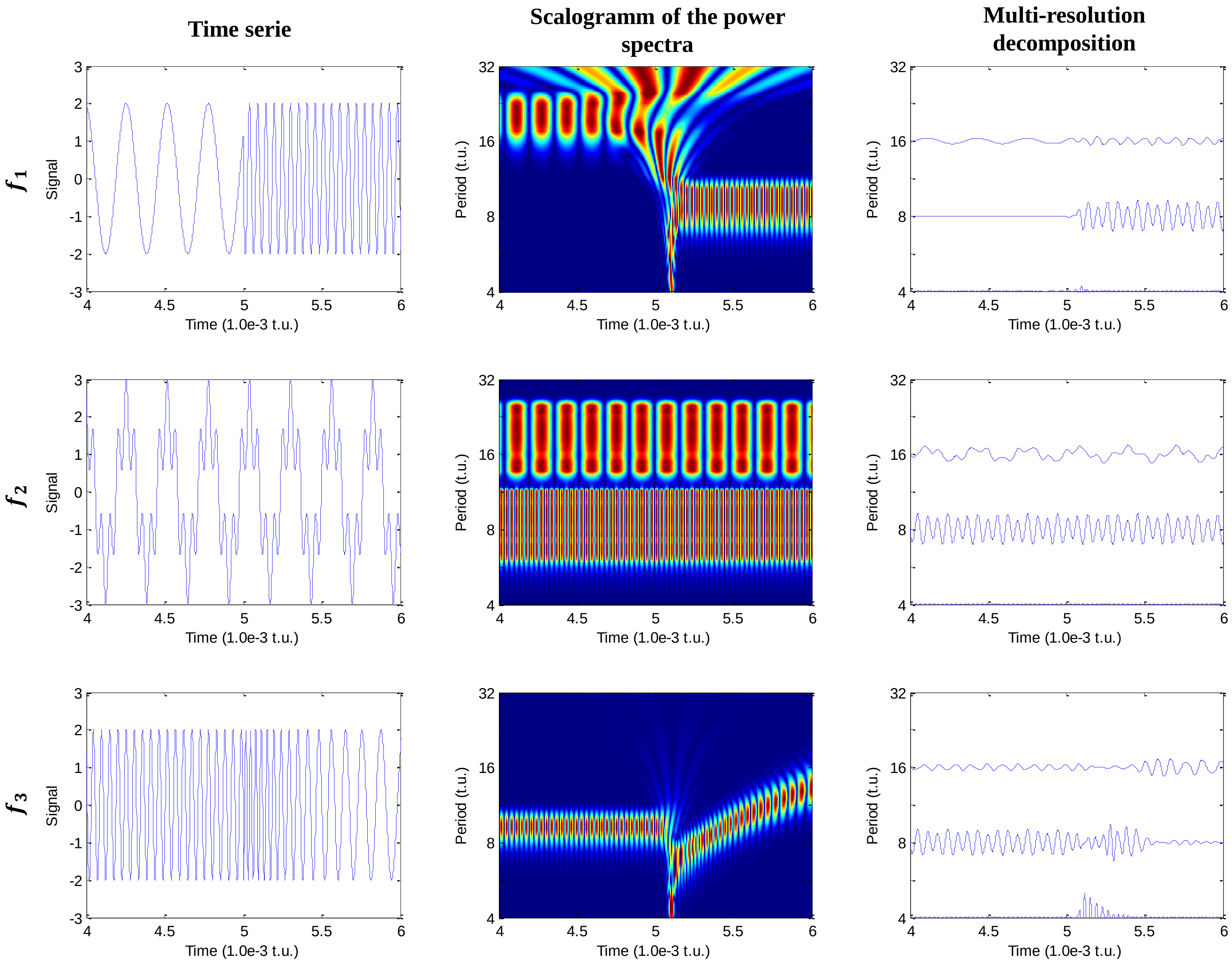
Time serie
Scalogramm of the power spectra
Multi-resolution decomposition
f_1
f_2
f_3
Signal
Period (t.u.)
Time (1.0e-3 t.u.)
3
2
1
0
-1
-2
-3
32
16
8
4
4.5
5
5.5
6

Figure S4:

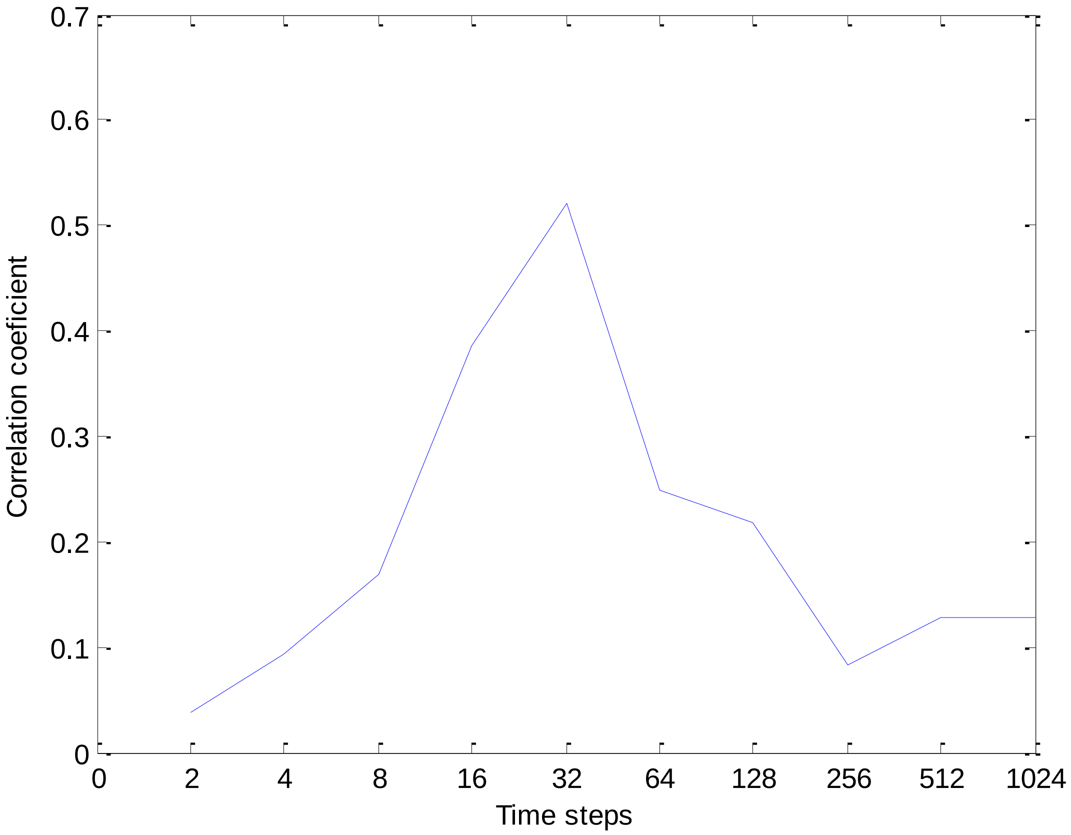

Figure S5:

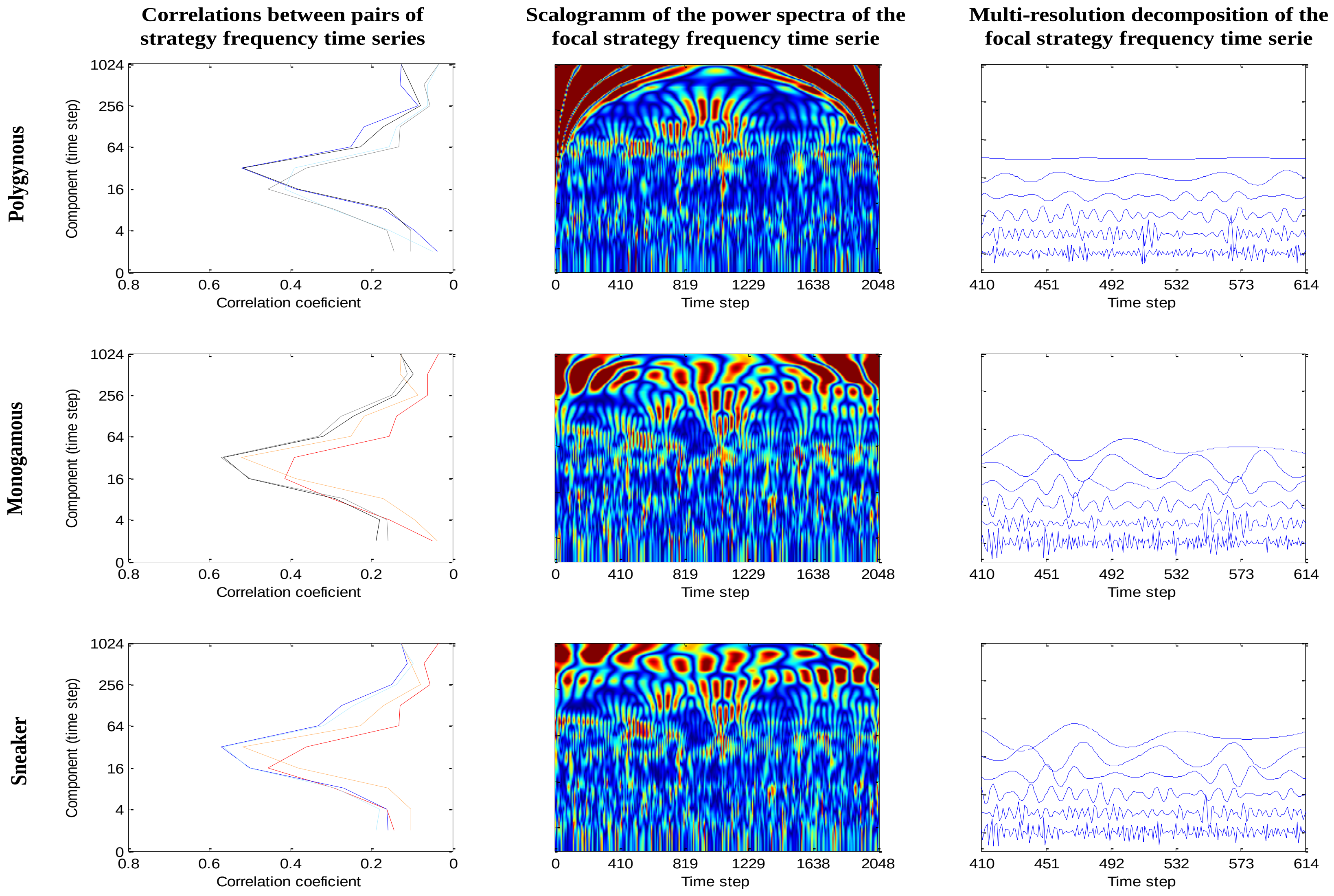

Correlations between pairs of strategy frequency time series
Scalogramm of the power spectra of the focal strategy frequency time serie
Multi-resolution decomposition of the focal strategy frequency time serie
Polygynous
Monogamous
Sneaker
Component (time step)
1024
256
64
16
4
0
0.8
0.6
0.4
0.2
0
Correlation coeficient
410
819
1229
1638
2048
Time step
451
492
532
573
614

References:

1 Legendre, S., Clobert, J., Moller, A. P. & Sorci, G. Demographic Stochasticity and Social Mating System in the Process of Extinction of Small Populations: The Case of Passerines Introduced to New Zealand. *The American Naturalist* **153**, 449-463 (1999).

2 Maynard-Smith, J. *Evolution and the theory of games*. (Cambridge University Press, 1982).

3 Sinervo, B. & Calsbeek, R. The developmental, physiological, neural, and genetical causes and consequences of frequency-dependent selection in the wild. *Annual Review of Ecology Evolution and Systematics* **37**, 581-610 (2006).

4 Sinervo, B. & Lively, C. M. The rock-paper-scissors game and the evolution of alternative male strategies. *Nature* **380**, 240-243 (1996).

5 Abarbanel, H. D. I. *Analysis of Observed Chaotic Data*. (Springer-Verlag, 1996).

6 Box, G. E. P. & Jenkins, G. M. *Time Series Analysis*. Revised edn, (Holden-Day, 1976).

7 Jenkins, G. M. & Watts, D. G. *Spectral Analysis and its Applications*. (Holden-Day, 1968).

8 Mandelbrot, B. *Fractales, hasard et finance*. (Flammarion, 1997).

9 Mari, J.-L., Glangeaud, F. & Coppens, F. *Traitement du Signal pour Géologues et Géophysiciens*. 460 (TECHNIP, 1997).

10 Meyer, Y. & Roques, S. *Progress in Wavelet Analysis and Applications*. (Frontieres, 1993).

11 Cazelles, B. *et al.* Wavelet analysis of ecological time series. *Oecologia* **156**, 287-304 (2008).

12 Shannon, C. E. A Mathematical Theory Of Communication. *Bell System Technical Journal* **27**, 379-423 (1948).
